# How Do Scores on the Functional Status Examination (FSE) Correspond to Scores on the Glasgow Outcome Scale-Extended (GOSE)?

**DOI:** 10.1089/neur.2021.0057

**Published:** 2022-03-04

**Authors:** Lindsay D. Nelson, Brooke E. Magnus, Nancy R. Temkin, Sureyya Dikmen, Geoffrey T. Manley, Steve Balsis

**Affiliations:** ^1^ Department of Neurosurgery and Neurology, Medical College of Wisconsin, Milwaukee, Wisconsin, USA.; ^2^ Department of Psychology and Neuroscience, Boston College, Chestnut Hill, Massachusetts, USA.; ^3^ Department of Neurological Surgery and Biostatistics, University of Washington, Seattle, Washington, USA.; ^4^ Department of Rehabilitation Medicine, University of Washington, Seattle, Washington, USA.; ^5^ Department of Neurological Surgery, University of California San Francisco, San Francisco, California, USA.; ^6^ Department of Psychology, University of Massachusetts Lowell, Lowell, Massachusetts, USA.

**Keywords:** functional limitations, Functional Status Examination, Glasgow Outcome Scale-Extended, item response theory, outcome measurement

## Abstract

This study was designed to determine how raw scores correspond between two alternative measures of functional recovery from traumatic brain injury (TBI), the Functional Status Examination (FSE) and the Glasgow Outcome Scale-Extended (GOSE). Using data from 357 persons with moderate-severe TBI who participated in a large clinical trial, we performed item response theory analysis to characterize the relationship between functional ability measured by the FSE and GOSE at 6 months post-injury. Results revealed that raw scores for the FSE and GOSE can be linked, and a table is provided to translate scores from one instrument to the other. For example, a FSE score of 7 (on its 0–21 scale, where higher scores reflect more impairment) is equivalent to a GOSE score of 6 (where GOSE is scaled on an 8-point scale, with higher scores reflecting less impairment). These results allow clinicians or researchers who have a score for a person on one instrument to cross-reference it to a score on the other instrument. Importantly, this enables researchers to combine data sets where some persons only completed the GOSE and some only the FSE. In addition, an investigator could save participant time by eliminating one instrument from a battery of tests, yet still retain a score on that instrument for each participant. More broadly, the findings help anchor scores from these two instruments to the broader continuum of injury-related functional limitations.

## Introduction

The Glasgow Outcome Scale-Extended (GOSE) and Functional Status Examination (FSE) are two well-recognized, leading, and validated interview-based measures of functional limitations after traumatic brain injury (TBI).^1–6^ A growing body of research indicates that these two instruments measure the same construct of injury-related functional limitations, implying one may be a reasonable substitute for the other.^5,7,8^ Each instrument has advantages, such as widespread recognition of the GOSE's 1–8 scale and the ability of the FSE, which has a wider score range, to more precisely characterize individual differences in TBI recovery.^5,8–14^ Given that these two leading instruments are used in research studies and clinical trials, there is a need to understand how scores on the two correspond.

Knowing how scores on the FSE and GOSE correspond would enable several research goals. For example, being able to translate GOSE to FSE scores and *vice versa* would allow researchers to combine data sets that administered only one of these measures. Or, an investigator who had limited time with a research participant might decide to administer just one instrument, while retaining information about how that participant would have scored on the other instrument. In this way, understanding how two instruments correspond may help with ongoing goals of developing an efficient yet precise outcome measurement for clinical trials.^15^ Recently, TBI researchers have turned their attention to advanced psychometric techniques, including item response theory (IRT), which allows for the linking of scores between measures by placing both measures on a common scale (a latent continuum, in this case injury-related functional impairment). Leveraging our team's recent work supporting the use of such models with the GOSE and FSE, we sought to link raw scores for each measure and create a table that researchers and clinicians can use to translate GOSE scores to FSE scores and *vice versa*. We also describe our approach to IRT score linking, which may be used to link other important instruments widely used in TBI research and clinical practice.

## Methods

### Participants

We performed secondary data analyses of patients with moderate-to-severe TBI who were enrolled in a randomized controlled trial of magnesium sulfate at Harborview Medical Center (Seattle, WA) between August 1998 and October 2004. This original study showed no positive effect of the magnesium sulfate intervention on outcomes, but the investigators found a negative effect of treatment in the lower-dosage group.^16^ For this original study, participants were required to have intracranial surgery within 8 h of injury, a post-resuscitation Glasgow Coma Scale (GCS)^17^ score of 3–12 or, if intubated, a GCS motor score of 1–5 without pharmacological paralysis. Patients who were under 14 years of age, unable to receive the study drug within 8 h of injury, who had serum creatinine concentrations >177 μmol/L, were pregnant, a prisoner, or who were living overseas were excluded from the analyses. Given the focus of the present study on 6-month measures of functional limitations, we analyzed data from the subset of participants (*n* = 357) who had sufficient outcome data at 6 months to be included in IRT models. The selection of this subsample is described further in Nelson and colleagues.^8^

### Outcome assessment

As described in previous publications using this data set,^16,18,19^ participants completed diverse neuropsychological assessments at various points in the first 6 months post-injury. Relevant to the current study, the GOSE and FSE were collected at 6 months post-injury. The GOSE was collected using the recommended interview guidelines in place at that time.^2^ In particular, questions about independence in and outside the home, work/school functioning, social functioning, psychological and relationship functioning, and other functional limitations were included. Responses to these questions were used to place patients on an 8-point ordinal scale: 1 = Death, 2 = Vegetative State, 3 = Lower Severe Disability, 4 = Upper Severe Disability, 5 = Lower Moderate Disability, 6 = Upper Moderate Disability, 7 = Lower Good Recovery, and 8 = Upper Good Recovery. For this analysis, we retained cases with scores that ranged between 2 and 8 inclusive because these are the outcome ranges for which FSE scores are formally defined.

The FSE structured interview was conducted with patient participants and/or their significant other (close family member or friend). Past work showed a strong correlation (0.80–0.82) between patient and significant other ratings^6,18^ and no IRT differential item functioning by respondent.^8^ The study used the original version of the FSE interview, which contained 10 subsections: Personal Care; Mobility/Ambulation; Mobility/Travel; Primary Activity (among work and school); Home Management; Leisure and Recreation; Social Integration; Cognitive & Behavioral Competency; Standard of Living; and Financial Independence. However, because the latter three domains were not retained in revisions of the instrument by its author, these domains were not included in the present analysis.^20^ Each domain on the FSE is rated on a 4-point ordinal scale, from 0 (same level of function as before injury) to 3 (complete or nearly complete reliance on others or inability to function in the domain), and these scores are summed to produce a total score reflecting the degree of injury-related functional impairment (range, 0–21 using seven domains, although persons who died were assigned an FSE score of 22 in the clinical trial). Both the FSE and GOSE interviews in this study included injury-related functional limitations regardless of whether their cause was TBI or other concurrent injuries.

### Statistical analyses

Analyses for the present study used IRT parameters established in a previous publication from this sample.^8^ This model entered the seven FSE items and the GOSE overall score into a single model to establish the relationship between these items and the latent continuum of injury-related functional limitations. In summary, the data met the two necessary assumptions for conducting IRT analyses—that the data represent one underlying dimension and are locally independent. For example, the first/second eigenvalue ratio from an exploratory factor analysis was sufficiently large (18.0), and the fit of a one-factor confirmatory factor analytical model adequate, to consider the data sufficiently unidimensional.^21,22^ A unidimensional IRT model was estimated using IRTPRO version 4.2 to quantify the *difficulty* (b) and *discrimination* (a) of FSE and GOSE items. In IRT terms, difficulty reflects the level of disability severity (the latent continuum; called *theta*) indicated by the item; discrimination reflects the strength of the relationship between each item and the latent continuum. (Higher discrimination means that the item yields more precise estimates of one's score on the latent continuum.)

As is described further in the Results, these item parameters were used to model the relationship between FSE and GOSE observed scores and theta scores and to perform single-group concurrent calibration (i.e., linking FSE to GSOE scores according to where they relate on the theta [latent disability] scale). We modeled our linking approach after one implemented in previous psychometric efforts.^23^

## Results

**FIG. 1.**
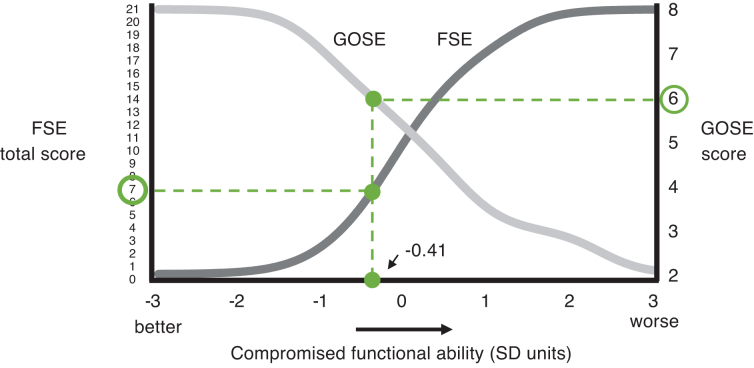
Correspondence between Glasgow Outcome Scale-Extended (GOSE) and Functional Status Examination (FSE) scores, according to their linkage along the latent continuum of injury-related functional disability. Note that the analysis only included persons who survived their injuries (GOSE, 2–8; FSE, 0–21).

IRT model parameters quantified the relationship between FSE and GOSE scores and the latent continuum of compromised functional ability. This model was used to plot the test characteristic curve (i.e., relationship between test scores and latent impairment level) for the FSE and GOSE across the continuum of functional impairment (Fig. 1) and develop a table linking GOSE to FSE scores (see Table 1). Figure 1 shows that as FSE scores increase from 0 to 21 (see left axis), the test characteristic curve for the FSE rises across the continuum of compromised functional ability (horizontal axis). This monotonically increasing curve is consistent with the design of the FSE, which characterizes greater disability with higher scores. In contrast, the equivalent curve for the GOSE is monotonically decreasing (from 8 to 2) because the GOSE characterizes greater disability with lower scores (see right axis in Fig. 1).

**Table 1.** Correspondence between GOSE and FSE Scores^a^

GOSE^b^	FSE	Theta
8	0	<−1.63
	1	–1.63 to 1.20
7	2	–1.19 to −0.97
	3	–0.96 to −0.79
	4	–0.78 to −0.65
	5	–0.64 to −0.53
	6	–0.52 to −0.42
6	7	–0.41 to −0.32
	8	–0.31 to −0.22
	9	–0.21 to −0.12
	10	–0.11 to −0.02
	11	–0.01 to 0.08
5	12	0.09–0.19
	13	0.20–0.31
	14	0.32–0.44
	15	0.45–0.59
4	16	0.60–0.75
	17	0.76–0.95
	18	0.96–1.17
	19	1.18–1.42
3	20	1.43–1.82
	21	1.83–2.36
2	21	≥2.37

Theta reflects the level of the latent construct of functional limitations modeled by item response theory (IRT), in standard deviation units.

^a^ Although FSE total scores (sum of item scores) are formally defined as ranging from 0 to 21, its authors have previously assigned persons who died (GOSE 1) an FSE score of 22.

^b^ GOSE score placed next to the FSE score for which it matched most closely in the model.

FSE, Functional Status Examination; GOSE, Glasgow Outcome Scale-Extended.

This latent variable (theta), compromised functional ability, provides a common scale on which to link the FSE and GOSE. In other words, each point along the continuum between −3.00 and 3.00 standard deviations (SDs) corresponds both to a point along the FSE curve *and* to a point along the GOSE curve. For example, note that a score of 7 on the FSE corresponds to a score of 6 on the GOSE, because each raw score corresponds to a value of −0.41 SDs of compromised functional ability. Figure 1 helps to illustrate the overall findings. In order to derive more precise/quantitative estimates of the relationship between the latent continuum, FSE, and GOSE scores, we created Table 1, which uses the IRT model parameters to translate the data reflected in Figure 1 into columns of values in Microsoft Excel (Microsoft Corporation, Redmond, WA), thereby establishing a value for the FSE and GOSE at every point along the theta continuum between −3.00 and 3.00, in increments of 0.01 SD units. Note that this produced estimated FSE and GOSE scores that were decimals.

With these values established, we traced the FSE scores from 0 to 21, breaking them into integer scores based on traditional rounding rules (e.g., estimated FSE 0.00–0.49 we coded as the integer score 0, estimated FSE between 0.50 and 1.49 coded as the integer score 1, all the way up to an FSE score of 21). These ranges of theta associated with each predicted FSE score are presented in Table 1. Then, GOSE scores were extracted from the point along the continuum closest to each raw FSE score (i.e., point closest to FSE score of 0, 1, 2, etc.; see Table 1). The resulting Table 1 provides a cross-walk of GOSE, FSE, and theta scores, which can be used by clinicians and researchers to determine how a patient's raw score on one instrument, say the FSE, corresponds to a score on the GOSE.

Note that because FSE item-level scores are not formally defined for persons who are dead, and this IRT model relied on item-level data, this model included living persons only (i.e., GOSE ≥2). The range of FSE scores derived from the model is 0–21 (see Fig. 1) because the model was based on its seven items (each scaled 0–3, where 3 reflects that a person is either not participating in an outcome domain or is completely dependent on support to do so, which can occur because of severe impairment or being in a vegetative state). This scaling, in turn, formally restricts the model-estimated maximum FSE score to 21. However, from Table 1, it is apparent that an FSE score of 21 could reflect either a GOSE 2 or 3, an assignment that would require clinical information to differentiate. This is because persons with a 21 FSE score were rated a 3 (maximum impairment) on all items, which could occur because of being in a vegetative state or otherwise being severely globally unable to participate in major life domains independently. Further, although the FSE summed score is not formally defined for persons who died, based on past recommendations one could assign an FSE score of 22 to persons with GOSE 1 (dead) on conceptual grounds.^16^

## Discussion

The GOSE and FSE are both respected measures of TBI-related functional limitations that have been used in clinical studies of TBI. Historically, formal guidance was not available to identify how scores on these instruments are related. The current study used a process of IRT linking to show how FSE and GOSE scores correspond to each other and in relation to the level of functional limitations reflected by different FSE and GOSE scores. We also described the method for IRT linking, which may provide a template for other attempts at linking measures important to TBI research. Further, we created a table that includes each score on the two measures and the corresponding score on the other test that indicates the same level of functional ability. As one would expect, our findings demonstrate that most GOSE scores reflect ranges of multiple possible FSE scores, meaning that the GOSE is a coarser representation of function than the FSE.

Researchers and clinicians can use the score conversion table to better leverage existing data and better characterize TBI patients. For example, the findings anchor GOSE and FSE scores to the level(s) of the broader continuum of functional limitations reflected by those scores. Importantly, the findings could be used to combine data sets from studies regardless of whether they administered the FSE or GOSE. In light of proposals to use the FSE as a more granular outcome measure than the GOSE for clinical trials,^8,24^ the findings could also inform how adopting the FSE as a primary outcome measure in clinical trials would work, as compared to traditional outcome measurement strategies (e.g., to identify how differing GOSE cutoffs for “favorable outcome” translate in FSE terms).

The relatively simple, straightforward method of IRT linking described here may prove useful to other aspects of TBI research. For example, IRT could be used to develop score conversion tables for other widely used instruments of mild TBI (mTBI)-related symptoms, quality of life, and other constructs or even to map instruments from differing constructs (e.g., cognitive performance, symptoms, or, potentially, biomarkers) to each other by charting where they fall on the latent continuum of TBI-related disability. The method described in the current study could be used any time the same sample of persons completes multiple measures, as is common in extended neuropsychological assessments. Beyond the scope of this article, IRT linking is a deep subspecialty with methods available for more complex study designs.

It is important to note that FSE-GOSE translations enabled by Table 1 reflect their linkage along the latent continuum of functional impairment, with potentially different meaning than traditional GOSE scores. For example, a GOSE score of 5 indicates that the patient can be left alone at home for 24 h, whereas this is not guaranteed by an IRT-predicted GOSE score. On the other hand, it is possible that IRT-based scores are more precise or valid than the traditional GOSE score because of being characterized according to the latent continuum of functional limitations based on all interview responses provided. Additional research would be useful to explicate the similarities and distinctions between traditional observed scores on these instruments and the latent continuum they can be considered to reflect.

This GOSE-FSE score conversion table significantly advances an understanding of how the GOSE and FSE measure different parts of the continuum of functional limitations while also explicitly mapping how their scores compare to each other. Note that because our model used impairment ratings provided irrespective of their source (TBI vs. peripheral injuries), we cannot verify that the score mapping would be equivalent for ratings of the specific impact of TBI on patients’ functioning. Similarly, this model may not translate to other subpopulations of TBI (e.g., mTBI) or time points beyond 6 months post-injury, given that these instruments and the resultant IRT model have not yet been demonstrated to be invariant over severity groups and time. However, with this type of approach, it would be theoretically possible to harmonize longitudinal data across these two instruments. In summary, this study demonstrates how sophisticated psychometrics can bridge gaps in knowledge about alternative measures important to TBI research and will help fuel continued advancements in modern outcome measurement approaches for clinical and translational studies of TBI.

## Authors' Contributions

Dr. Nelson contributed to the conceptualization of the study, data analysis, interpretation of the findings, and drafting of the manuscript. Dr. Magnus contributed to the conceptualization of the study, interpretation of the findings, and critically reviewed and approved the manuscript. Dr. Temkin contributed to data analysis decisions and critically reviewed and approved the final manuscript. Dr. Dikmen critically reviewed and approved the final manuscript. Dr. Manley critically reviewed and approved the final manuscript. Dr. Balsis contributed to the conceptualization of the study, data analysis, interpretation of the findings, and drafting of the manuscript.

## Abbreviations Used

FSE: Functional Status Examination
GCS: Glasgow Coma Scale
GOSE: Glasgow Outcome Scale-Extended
IRT: item response theory
mTBI: mild TBI
SDs: standard deviations
TBI: traumatic brain injury
